# Plant Abiotic Stress

**DOI:** 10.1155/2013/432836

**Published:** 2013-10-01

**Authors:** Ji Huang, Alexander Levine, Zhoufei Wang

**Affiliations:** ^1^State Key Laboratory of Crop Genetics and Germplasm Enhancement, Nanjing Agricultural University, Nanjing 210095, China; ^2^Institute of Life Sciences, The Hebrew University of Jerusalem, Givat Ram Campus, 91904 Jerusalem, Israel

Abiotic stress such as cold, drought, salt, and heavy metals largely influences plant development and crop productivity. Abiotic stress has been becoming a major threat to food security due to the constant changes of climate and deterioration of environment caused by human activity. To cope with abiotic stress, plants can initiate a number of molecular, cellular, and physiological changes to respond and adapt to such stresses. Better understanding of the plant responsiveness to abiotic stress will aid in both traditional and modern breeding applications towards improving stress tolerance. Studies on some special wild plant species with high stress tolerance also greatly contribute to our understanding of stress tolerance.

With the development of modern molecular biology, genomics approaches have been applied in crop breeding while not popularly in practice. In this special issue, B. A. Akpınar et al. present a compressive review on genomic approaches, including structure genomics, comparative genomics, and functional genomics, for crop improvement against abiotic stress. Some traditional and modern genomics approaches of crop improvement, such as expressed sequence tag (EST) profiling, microarray, Targeting Induced Local Lesions IN Genomes (TILLING), and next generation sequencing (NGS), are summarized in this review. In the other review paper, H. Budak et al. focused on the studies of drought tolerance in modern and wild wheat. They reviewed recent advances on drought related gene/QTL identification, studies on drought related molecular pathways, and current efforts on the improvement of wheat cultivars for drought tolerance.

The exploitation and utilization of crop germplasm resources are the basis of crop breeding. However, with continuous collection of germplasm resources, the size of populations has been becoming bigger, which hinders the evaluation and utilization of the germplasm resources. Constructing core collection is an efficient way to solve the problem. A core collection is a representative sample of the whole collection which has minimum repetitiveness and maximum genetic diversity of a plant species. J. Wang et al. suggest a strategy to construct a core collection based on drought resistant germplasm resources in wheat. The results showed that the strategy was effective and was valuable in another crop's core collection construction. 

The wild plant species with high tolerance to abiotic stress have attracted more and more attentions of plant biologists, especially when the studies within a crop species on improving the stress tolerance show the limitation. Understanding the mechanisms for stress tolerance achieved by these wild species will help in crop improvement; even some relatives can be directly or indirectly applied in crop breeding by cytological ways. H. Budak et al. introduce the research advances on wild emmer wheat which is important for its high drought tolerance. W. Liu et al. present cold induced physiological responses of naked oats (*Avena nuda* L.), a cold-tolerant plant species, and show that the cold tolerance involves the increase of antioxidant activities of several reactive oxygen species (ROS) savaging enzymes. The heavy metal contamination is an environmental problem in the margin sea. H.-p. Jiang et al. studied the responses of green algae species *Ulva prolifera* and *Ulva linza* to Cd^2+^ and concluded the major physiological parameters involved in the Cd^2+^ adaptation. 

It is necessary to further clarify the mechanisms underlying plant stress responses through modern biological technologies, especially to understand stress responses of some wild plant species with extremely high stress tolerance, which will be eventually applied in developing crops with high stress tolerance.

*Ji Huang* *Ji Huang* *Alexander Levine* *Alexander Levine* *Zhoufei Wang* *Zhoufei Wang*

